# First‐Line Tocilizumab Monotherapy for Juvenile Systemic Sclerosis With Interstitial Lung Disease: A Case Report

**DOI:** 10.1155/crrh/2914024

**Published:** 2026-08-12

**Authors:** Tadafumi Yokoyama, Natsumi Inoue, Satoshi Watanabe, Takashi Matsushita, Taizo Wada

**Affiliations:** ^1^ Department of Pediatrics, Kanazawa University Hospital, Takara-Machi 13-1, Kanazawa Ishikawa, 920-8640, Japan, kanazawa-u.ac.jp; ^2^ Respiratory Medicine, Kanazawa University Hospital, Takara-Machi 13-1, Kanazawa Ishikawa, 920-8640, Japan, kanazawa-u.ac.jp; ^3^ Department of Dermatology, Kanazawa University Hospital, Takara-Machi 13-1, Kanazawa Ishikawa, 920-8640, Japan, kanazawa-u.ac.jp

**Keywords:** early-stage, interstitial lung disease, juvenile systemic sclerosis, pediatric, tocilizumab

## Abstract

Systemic sclerosis, a rare autoimmune disease in children, frequently presents with aggressive clinical features and high risk of interstitial lung disease. Tocilizumab, an anti–interleukin‐6 receptor monoclonal antibody, has exhibited efficacy in adult systemic sclerosis with interstitial lung disease; however, evidence in pediatric cases remains extremely limited. We report a 12‐year‐old Japanese girl who developed early‐stage juvenile systemic sclerosis with interstitial lung disease, presenting with Raynaud’s phenomenon, puffy fingers, positive anti–topoisomerase I antibody, reduced pulmonary function, and ground‐glass opacities on high‐resolution computed tomography. Consequently, tocilizumab monotherapy was initiated. During a follow‐up period of over 3 years, her pulmonary function remained stable, and high‐resolution computed tomography showed partial resolution of interstitial changes. No significant adverse events, such as severe infections or cytopenias, were observed. Early tocilizumab initiation may represent a potential therapeutic option for juvenile systemic sclerosis with interstitial lung disease. However, further studies are warranted to validate its role and optimize treatment guidelines for this rare but severe condition.

## 1. Introduction

Systemic sclerosis (SSc) is a rare, chronic autoimmune disease characterized by vasculopathy, immune dysregulation, and progressive skin and internal organ fibrosis [1]. Interstitial lung disease (ILD) is one of its most severe complications, affecting up to half of patients and is a leading cause of morbidity and mortality [1]. Once pulmonary fibrosis is established, treatment response is limited, highlighting the importance of early recognition and timely therapeutic intervention [2].

Juvenile SSc (JSSc) follows a more aggressive clinical course and is frequently associated with visceral involvement, including ILD [3]. Tocilizumab (TCZ), a humanized monoclonal antibody that targets the interleukin‐6 (IL‐6) receptor, has recently emerged as a therapeutic option for SSc‐ILD [2, 4, 5]. Data on JSSc‐associated ILD (JSSc‐ILD) are extremely limited; however, some case reports have reported favorable outcomes with TCZ in children with refractory JSSc, where conventional therapies had failed [6].

We report the case of a 12‐year‐old girl with early‐stage JSSc‐ILD who was successfully treated with TCZ monotherapy, without concomitant immunosuppressants, highlighting the effectiveness and safety of TCZ for early‐stage JSSc‐ILD, while avoiding additional immunosuppressive therapy.

## 2. Case Presentation

A 12‐year‐old Japanese girl with no significant past or family medical history was referred to our hospital for the evaluation of Raynaud’s phenomenon and a positive antinuclear antibody (ANA) test. Two years before presentation, she had experienced recurrent episodes of finger discoloration that resembled chilblains during cold weather, which resolved spontaneously. Several months before referral, she developed progressive fatigue, exertional dyspnea, and a dry cough. She reported no heartburn after meals. In the summer, she noted sudden coldness and purplish discoloration of all toes. Laboratory testing revealed an ANA titer of 1:320, and the patient was referred to our hospital for further evaluation.

**FIGURE 1:**
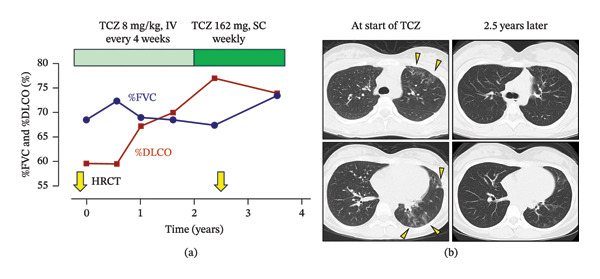
Clinical course of the patient. (a) Longitudinal changes in pulmonary function. The graph shows changes in %FVC (blue) and %DLCO (red) over time. Throughout the clinical course, %FVC remained stable, whereas %DLCO showed an overall improvement. This improvement was sustained even after switching to subcutaneous TCZ administration. (b) Computed tomography of the patient’s lung. The ground‐glass opacities (yellow arrowheads) observed on chest high‐resolution computed tomography improved after 2.5 years of TCZ treatment. Alt text: Figure showing the patient’s clinical course during TCZ therapy. Panel (a) shows longitudinal pulmonary function tests, demonstrating stable %FVC and an overall improvement in %DLCO that was sustained after switching from intravenous to subcutaneous TCZ. Panel (b) shows chest HRCT images demonstrating ground‐glass opacities in the left lung at TCZ initiation and partial resolution after 2.5 years of treatment.

Physical examination revealed puffy fingers and dilated and reduced nailfold capillary loops, without skin thickening (modified Rodnan skin score: 0). Her oxygen saturation was 99% on room air, and auscultation revealed no lung crackles. Laboratory findings demonstrated a leukocyte count of 5420/μL (neutrophils: 48.1%, lymphocytes: 40.0%), hemoglobin of 12.4 g/dL, platelet count of 28.4 × 10^4^/μL, and normal complement levels. Inflammatory markers were low (C‐reactive protein: 0.03 mg/dL, IL‐6: 1.5 pg/mL, erythrocyte sedimentation rate: 8 mm/h, immunoglobulin G: 1482 mg/dL). Immunological testing revealed anti–topoisomerase I antibody positivity (288.0 U/mL) and anti‐RNP, anti‐Sm, anti–SS‐A, anti–SS‐B, anti‐ssDNA, and anti‐dsDNA antibody negativity. Serum KL‐6 and SP‐D were normal (234 U/mL and 50.4 ng/mL, respectively). Urinalysis was normal. Pulmonary function testing revealed a decreased percentage of predicted forced vital capacity (FVC; %FVC: 68.5) and diffusing capacity of the lung for carbon monoxide (%DLCO: 59.5) (Figure 1a). Echocardiography demonstrated no pulmonary hypertension. High‐resolution computed tomography (HRCT) revealed ground‐glass opacities along the bronchi in the left lower and upper lung fields (Figure 1b). The abnormalities were considered a ground‐glass‐predominant, unclassifiable interstitial pattern rather than a definite NSIP pattern. These abnormalities persisted despite a 14‐day tosufloxacin treatment.

She fulfilled the 2013 American College of Rheumatology/European League Against Rheumatism classification criteria for SSc, scoring 12 points (puffy fingers: 2, nailfold capillary changes: 2, ILD: 2, Raynaud’s phenomenon: 3, SSc‐specific antibody: 3) [7]. A diagnosis of JSSc‐ILD was established [7]. Intravenous TCZ (8 mg/kg; every 4 weeks) treatment was initiated to prevent ILD progression, adjusting the dosage according to body size. The regimen was switched to subcutaneous TCZ (162 mg; weekly) after 2 years of treatment. During the early treatment period, she occasionally experienced a dry cough, which gradually decreased in frequency. Pulmonary function testing at 3 years demonstrated stable %FVC (73.5) and improved %DLCO (74.0). Follow‐up HRCT performed after 2.5 years revealed partial improvement of the interstitial abnormalities (Figure 1).

Throughout the clinical course, the mRSS remained 0 at baseline and at 36 months, and neither sclerodactyly nor proximal skin thickening developed during follow‐up. The absence of skin fibrosis throughout the disease course was consistent with a JSSc sine scleroderma phenotype. Additionally, gastroesophageal reflux symptoms did not develop. Complement C4 was reduced after TCZ initiation; however, C3 and CH50 remained within normal ranges. During the 3 years of treatment, she developed no severe infections, agranulocytosis, or hypogammaglobulinemia.

## 3. Discussion

Our patient represents the rare pediatric case in which TCZ monotherapy stabilized JSSc‐ILD for several years. Pulmonary function was preserved, radiological abnormalities partially improved, and treatment was well tolerated without serious adverse events such as severe infections, cytopenias, or hypogammaglobulinemia. Disease control was achieved without concomitant immunosuppressants, underscoring the potential of TCZ as a standalone therapeutic option. The transition from intravenous to subcutaneous administration further reduced the treatment burden and enabled the patient to maintain normal school activities.

In recent years, SSc‐ILD treatment strategies in adults have advanced significantly. Traditional immunosuppressive agents such as cyclophosphamide and mycophenolate mofetil have been administered as first‐line therapies; however, their efficacy remains modest, with their long‐term safety being concerning [2, 8]. Between 2016 and 2019, targeted therapies, including nintedanib and TCZ, have been established [4, 9]. TCZ has demonstrated efficacy in attenuating the decline of FVC, particularly in early‐stage SSc‐ILD. The Phase II faSScinate trial indicated a trend toward lung function preservation, and the subsequent Phase III focuSSced trial confirmed that TCZ significantly attenuated the decline in FVC over 48 weeks in patients with SSc‐ILD, leading to its regulatory approval for this indication in adults [4, 5]. These findings suggest that TCZ may be beneficial when initiated before irreversible pulmonary fibrosis develops [4, 5].

However, evidence on the management of JSSc‐ILD remains extremely limited, primarily due to the disease’s rarity and the paucity of large‐scale clinical trials [3]. In adults, conventional immunosuppressants are commonly employed; however, no agent has demonstrated consistent efficacy or safety specifically in JSSc‐ILD [3]. Furthermore, the potential gonadotoxicity of cyclophosphamide and adverse effects associated with long‐term conventional immunosuppression may be particularly concerning in adolescents [10].

Reports of TCZ administration in JSSc‐ILD are scarce and have largely described standard immunosuppressive therapy–unresponsive, refractory cases [6]. However, JSSc is often associated with more aggressive phenotypes, and ILD represents one of the most serious complications, impairing pulmonary function, decreasing quality of life, and increasing mortality [1, 3]. Once pulmonary fibrosis is established, the treatment response is limited, emphasizing the importance of early diagnosis and timely therapeutic intervention to prevent irreversible lung damage and long‐term functional impairment [1, 2].

In our case, at the time of treatment initiation, therapeutic options including MMF and cyclophosphamide were discussed. Current EULAR recommendations state that MMF, cyclophosphamide, or rituximab should be considered for the treatment of SSc‐ILD, while TCZ should also be considered based on evidence from randomized controlled trials [11]. However, these recommendations are based predominantly on adult populations, and no established treatment hierarchy exists for JSSc‐ILD.

The patient had symptomatic, early‐stage ILD characterized by reduced pulmonary function, anti–topoisomerase I antibody positivity, and ground‐glass‐predominant HRCT abnormalities without established fibrotic changes. Following multidisciplinary discussion involving pediatricians, pulmonologists, and dermatologists, as well as shared decision‐making with the patient and her parents, off‐label TCZ monotherapy was selected. The patient preferred monthly intravenous treatment over daily oral medication and wished to avoid broad immunosuppression because of concerns about its potential adverse effects. This decision reflected the patient’s preferences and concerns and was not based on evidence that MMF causes more adverse events in children.

Unlike patients enrolled in the faSScinate and focuSSced trials, our patient had neither elevated acute‐phase reactants nor diffuse cutaneous involvement. Therefore, direct extrapolation from these adult trials is uncertain. However, a post hoc analysis of the focuSSced trial found that baseline serum IL‐6 elevation was not predictive of response to TCZ, whereas earlier disease duration and anti–topoisomerase I antibody positivity appeared to be associated with a greater apparent treatment effect [12]. Normal KL‐6 and SP‐D levels in our patient may have reflected the limited radiographic extent of the early lung abnormalities rather than the absence of ILD activity. Nevertheless, the contribution of TCZ cannot be distinguished from spontaneous stability in this single case.

No major adverse events were observed throughout the course of TCZ treatment; however, a reduction in complement C4 was noted, whereas C3, CH50, urinalysis, and anti‐dsDNA antibody findings remained normal. TCZ‐associated reductions in complement levels have been reported and may reflect decreased IL‐6–dependent hepatic synthesis [13]. In our patient, the isolated mild reduction in C4 was not accompanied by clinical features of immune‐complex disease or serious infection and did not require treatment modification. However, its long‐term clinical significance remains uncertain.

No established TCZ dosing regimen exists for JSSc‐ILD. We initially administered intravenous TCZ at 8 mg/kg every 4 weeks, extrapolating from pediatric rheumatologic dosing. At Month 24, when the patient was 13 years old and weighed 45.0 kg, treatment was changed to subcutaneous TCZ at 162 mg weekly, corresponding to the adult SSc‐ILD regimen. The switch was made primarily to reduce hospital visits and interference with school attendance after clinical stability had been confirmed. No abrupt change in symptoms or pulmonary function was observed after the switch. However, this single case cannot define the optimal timing for switching formulations; the decision was individualized according to disease stability, body size, ability to self‐administer, and patient preference.

Although further studies are required to establish the long‐term efficacy and safety of TCZ monotherapy in JSSc‐ILD, our case supports its potential as a therapeutic option, particularly when initiated in early disease stages.

## Author Contributions

Tadafumi Yokoyama and Taizo Wada designed the study. Tadafumi Yokoyama, Natsumi Inoue, Satoshi Watanabe, and Takashi Matsushita treated the patient. Tadafumi Yokoyama and Natsumi Inoue wrote the manuscript. Tadafumi Yokoyama, Natsumi Inoue, and Taizo Wada critically reviewed the manuscript and supervised the whole study process.

## Funding

We received no honorarium, grant, or other forms of payment to produce this manuscript.

## Disclosure

All authors read and approved the final manuscript.

## Ethics Statement

According to the institutional policy of Kanazawa University Hospital, ethical review was not required for this single‐patient case report.

## Consent

In reporting this case, assent was obtained from the patient, and written informed consent was obtained from the parents.

## Conflicts of Interest

The authors declare no conflicts of interest.

## Supporting Information

Additional supporting information can be found online in the Supporting Information section.

## Supporting information


**Supporting Information** CARE checklist.

## Data Availability

Data sharing does not apply to this article because no datasets were generated or analyzed in this case report.
